# Effectiveness of multi-component modular intervention on screen-based and non-screen-based sedentary time among adolescents in an urban area of Mangalore: a school-based cluster randomised controlled trial-protocol

**DOI:** 10.12688/f1000research.142350.2

**Published:** 2024-03-11

**Authors:** Soundarya Janani S, Nithin Kumar, Mithun Rao, Rekha T, Prasanna Mithra, Bhaskaran Unnikrishnan, Ramesh Holla, Saraswathy M Vikraman, Himani Kotian

**Affiliations:** 1 Department of Community Medicine, Kasturba Medical College, Mangalore, Manipal Academy of Higher Education, Manipal, India

**Keywords:** Adolescent, Screen time, Sedentary behaviour, Modular intervention, Cluster randomized control trial.

## Abstract

**Background:**

Behavioural risk factors may often present during adolescence and account for 70% of premature deaths during adulthood. Excessive sedentary behaviour and screen time have become significant concerns, especially among adolescents, due to their potential negative impact on physical and mental health. Adolescents with a high screen-based sedentary time are more likely to be physically inactive, have unhealthy body structure and poor academic performance. The objective of our study is to assess the effect of multi-component modular educational intervention on screen-based sedentary time (SST) and non-screen-based Sedentary time (NSST) among adolescents.

**Methods:**

Ethical approval for the study has been obtained from the institutional Ethics Committee of Kasturba Medical College in Mangalore, India. This cluster randomized control trial will be carried out in schools located in the urban area of Mangalore. Using simple randomization, the eligible schools will be randomized into intervention and control arms, each consisting of 10 clusters. A multi-component modular educational intervention will be administered to participants in the intervention group at baseline, second and fourth month. The control group will receive the standard curriculum. Both the groups will be assessed at baseline and at second month, fourth month and sixth month of follow up for SST, NSST and level of physical activity. Anthropometric measurements like height, weight, waist circumference and hip circumference will be taken at baseline and sixth month of follow up.

**Results:**

A comprehensive school-based modular educational intervention can have cumulative advantages by reducing screen- and non-screen-based sedentary time, and encouraging physical activity. Similar modular teaching can be incorporated into the curriculum, which will promote healthy life-style among the adolescents.

## Introduction

Non-communicable diseases (NCDs) according to the World Health Organization (WHO) are long-lasting, non-infectious conditions with slow progression that are brought on by a confluence of genetic, physiological, environmental, and behavioral variables. ^1^ Around 41 million deaths, viz seventy-four per cent of total deaths worldwide each year are attributed to NCDs. This number includes 14 million premature deaths from NCDs, the bulk of which are preventable. ^2^ By 2030, it is anticipated that 55 million people will die annually from NCDs if immediate and appropriate interventions are not made. ^3^

Behavioural factors including lack of physical activity (PA) and unhealthy diet result in metabolic conditions like weight gain and obesity, high blood pressure, high cholesterol, and high blood sugar levels which are the main risk factors for NCDs. Rapid globalization and urbanization have resulted in the spread of unhealthy diets and poor lifestyles worldwide. ^4^ Given that these behavioural factors contribute significantly to the development of the majority of non-communicable diseases (NCDs), their effects on health across the lifespan of an individual, especially during adolescence have gained considerable attention in recent times. ^5^

According to the WHO, adolescents are young people, aged 10 to 19, who are rapidly developing in terms of their bodies, minds, and social connections. ^6^ It is also a crucial period for the establishment of health-related behaviours and lifestyle choices. Physical inactivity, substance use, unhealthy diet, being overweight and obesity are all risk factors for NCDs which are not seen as potential threats during adolescence. However, these risk factors that begin during adolescence account for 70% of premature deaths during adulthood. ^7^

In response to the WHO Global Action Plan for the Prevention and Control of NCDs, India developed a National Action Plan to reduce the number of premature deaths due to NCDs. ^8^ However, until recently the discussion around NCD prevention strategies mostly overlooked adolescents and focused only on the adult population. ^9^

Research involving sedentary time and sedentary behaviour has gained importance in recent times. ^10^ Evidence demonstrates a strong link between excessive sedentary behaviour and poor health outcomes. Sedentary behaviour as an independent entity has drawn a lot of attention as a risk factor for poor physical and mental health outcomes in children and adults. ^11^

**Sedentary behaviour (SB)** is any waking behaviour that involves sitting, reclining, or lying down while spending less than 1.5 metabolic equivalents of task (MET) of energy. It is seen in various domains in an adolescent’s life at home, at school, while using transport, and during free time. Examples include sitting in the school bus, playing board games, watching television, reading while sitting or lying down, and sitting in the classroom. ^10^ Excessive sedentary behaviour and screen time have become significant concerns, especially among adolescents, due to their potential negative impact on physical and mental health.

**Screen time (ST)** refers to the amount of time an individual spends engaged with screens, such as watching television, using computers, playing video games, or using smartphones and tablets.

**Screen-based sedentary time (SST)** is the amount of time spent using a screen-based device while being sedentary in any setting (e.g., school, home, or recreation). Examples of such devices include smartphones, tablets, computers, and televisions. SST, and in particular the time spent viewing television, is thought to be a key proxy for sedentary behaviour in adolescents. ^11^

Sedentary behaviour and screen time are closely related since screens often serve as the platform for various sedentary activities. The ease of accessibility and its utility for both academics and entertainment has led to a rise in screen time among children and adolescents. The recent COVID-19 pandemic led to significant changes in various aspects of our lives, including time spent using screens. Online classes and social isolation brought on by frequent lockdowns during the pandemic have also led to an increase in screen time among children and adolescents. ^12^ Various research studies have generated evidence indicating the relation between excessive screen time and the development of depression, anxiety, inattention, poor sleep, and physical inactivity among children and adolescents. ^13^

**Non-screen-based sedentary time (NSST)** is time spent engaging in sedentary activities, that don’t include the use of a screen such as doing paper-based homework, reading a physical book, playing board games and time spent on car rides and studying. ^11^

As per the recommendations by WHO and the American Academy of Paediatrics (AAP), children under the age of two should not spend any time seated in front of a screen (such as watching TV or videos or playing computer games), and children between the ages of two and five should restrict their screen time to no more than one hour (less is ideal). ^14^ Adolescents who watch TV, use computers, or play video games for more than two hours a day (screen-based sedentary time) are more likely to have an unhealthy body structure, poor fitness, and poor academic performance. Despite this, more than 30% of adolescents in the majority of countries around the world engage in screen-related behaviour that lasts more than two hours per day. ^15^

According to the WHO’s recommendation on sedentary behaviour, children and adolescents should limit their time spent sitting, especially screen time for leisure. ^16^

Over the past ten years, considerable research interest has been generated due to the rise in screen time use and its detrimental effect on health. As a result, intervention studies targeting a variety of demographic groups and contexts have been conducted to reduce screen-based sedentary time. ^17^^–^^20^

Interventional studies to reduce screen time targeting adolescents in schools in various Western and Latin American countries have been shown to bring about a significant reduction. The interventions applied in these studies can be categorized into three distinct groups: interventions consisting of motivational/volitional components such as health education sessions on healthy lifestyle, interventions using mass media and other health promotion materials like posters, and newsletters, and interventions involving modification of physical environments like standing desks and policy modifications such as curriculum changes. ^21^

This study aims to decrease screen-based and non-screen-based sedentary time in school-going teenagers through a multi-component modular educational intervention. Our study will indirectly focus on the third Sustainable Development Goal (SDG) ‘Good Health and Well-Being’ which aims to promote mental health and wellbeing while reducing premature mortality from noncommunicable diseases by one-third through prevention and early treatment.

### Hypothesis

Adolescents who receive a multi-component intervention targeting screen time and sedentary behaviours will show a significant reduction in screen sedentary time (SST) and Non screen sedentary time (NSST) compared to those who do not receive the intervention.

### Knowledge gaps

Reducing screen time among school-aged children is an important public health goal, as excessive screen time has been associated with various negative health outcomes, including obesity, sleep problems, and reduced physical activity. School-based interventions can play a critical role in addressing this issue.

However, a considerable knowledge gap still exists in this area. While some school-based interventions have shown promise in reducing screen time among students, there may be a lack of comprehensive understanding of the most effective strategies. Research is needed to identify whether the combination of interventions is successful in a school settings. There has been a limited number of school-based intervention studies for adolescents to decrease screen and non-screen-based sedentary time using a multi-component approach.

It may be regarded as an effective method to encourage adolescents to self-regulate their screen use. However, there hasn’t been much experimental research in India looking at the effects of such interventions The current study aims to close a gap in the body of knowledge on the effectiveness of a multi-component modular intervention on sedentary behaviour involving screens and other devices in urban Mangalore’s teenage populations.

### Literature review

In a school-based cross-sectional study in Brazil carried out among 2874 high school students in the age group between 14 and 19 years, the prevalence of high screen time was 79.5%. A higher proportion of males were engaged in high screen time compared to female students (84.3% vs.76.1%). On multivariate analysis, students belonging to high socio-economic status had higher odds of prolonged screen exposure. The level of physical activity and nutritional status was not found to be associated with high screen time among adolescents in the study. ^22^

In a cross-sectional study in Mumbai among 772 school-going adolescents in the age group between 10-15 years, screen time was high among girls compared to boys (218.21 min/d vs.165.3 min/d, p < 0.001). The combined prevalence of being overweight and obesity was 38.3%, low physical activity (PA) 38% and ST >120min/d was 85%. Clustering of high ST and low PA was seen in 69.2%. The odds of being overweight or obese in boys reporting low PA were 2.10 times higher, while the odds were 4.13 times higher in those with low PA+ST > 120 min/d. The study concluded that integrated interventions are necessary to allow greater reductions in obesity risk behaviours. ^23^

In a cross-sectional study in China among 971 children, it was observed that children typically spent one hour per day engaging in screen-based sedentary activity (SST) and one hour per day engaging in non-screen-based activity (NSST). Children were more likely to be overweight if they spent more time in SST. There was no connection between time spent on the NSST and overweight children (P > 0.05). This study demonstrated no link between NSST and being overweight, however, a correlation was observed between children who spent more time in SST and their likelihood of becoming overweight. ^13^

In a community-based cross-sectional study in New Delhi among 10 to 19-year-olds, the average daily screen time was calculated to be 3.8 hours. The majority of screen time—2.8 hours per day—is spent watching television. 68% of the teenagers admitted to watching television for more than the stipulated two hours a day. ^24^

In a cluster-randomised trial carried out among 8 ^th^ and 9 ^th^ graders of 20 urban schools in Cuenca-Ecuador, behavioural intervention was administered in two stages. Stage one of the intervention included diet, physical activity and screen time, while stage two included only diet and physical exercise. The screen time was evaluated at baseline, at 18 months for stage one and 28 months for stage two. At the end of the 18 ^th^ month of follow-up, lower TV time during weekday and weekends, and lower total screen time during weekday was observed among the intervention group. However, at the end of the 28 ^th^ month of follow-up, the TV time and total screen time on weekdays increased in the intervention group. The study concluded that the intervention to reduce screen time worked only if the intervention was specifically targeted at reducing screen time. ^17^

In an RCT carried out among 251 children aged 9 to 12 years in New Zealand, 127 children received intervention for 20 weeks which included training and educating the parent/caregiver on various strategies to reduce screen time. Screen time (TV) monitoring devices, mobility exercises, and support via online and newsletter were some of the interventions. The intervention group had more children participate in moderate-intensity physical activity (24.3 min/d; 95% CI: 0.94, 49.51; p = 0.06). No significant difference was observed in the BMI z score (−0.016; 95% CI: −0.084, 0.051; p = 0.64). The study concluded that home intervention to decrease screen time during leisure did not significantly reduce screen time or BMI. ^18^

A school-based cluster randomized control trial was conducted among 361 adolescents in the age group between 12-14 years in Australia which involved a 20-week intervention program. It included pedometers for self-monitoring physical activity, a smartphone app, a website, interactive seminars led by researchers, fitness equipment provided to schools, teacher-led physical education classes, lunchtime physical activity mentoring sessions, and newsletters for parents about screen time. The recreational screen time was recorded at baseline, 8 ^th^ month and 18 ^th^ month of intervention. Self-motivated screen time was significantly reduced by the intervention, while parental screen time guidelines were unaffected. ^19^

In an umbrella review that included systematic reviews and meta-analyses of interventions among children and adults directed at reducing sedentary behaviour (screen time, sitting time, or sedentary time), the interventions were found to be more successful at reducing sitting time than either physical activity alone or physical activity and sedentary behaviour combination interventions. There were slight but significant differences in viewing time in six out of the eleven meta-analyses (reported in seven studies). ^20^

### Aim

To determine the effectiveness of a multi-component modular intervention on screen-based sedentary time and non-screen-based sedentary time in school-going adolescents in an urban area of Mangalore.

### Objectives

1. To measure the screen sedentary time and non-screen sedentary time among the adolescent population.
2. To develop and implement a multicomponent modular intervention to reduce screen sedentary time and non-screen sedentary time among the adolescent population.
3. To determine the effectiveness of the multi-component modular intervention on screen-based and non-screen-based sedentary time among adolescents.

## Methods

### Study area

Mangalore is a major port city and the commercial headquarters of the district of Dakshina Kannada in the south Indian state of Karnataka. In comparison to the national average of 85%, the district has a high literacy rate of 93.4%. ^25^ Mangalore is a leading educational hub owing to the plethora of educational institutions offering high-quality education. This interventional study will be carried out in the schools located in the urban area of Mangalore.

### Study design

This will be a cluster randomized control trial. Consolidated Standards of Reporting Trials (CONSORT) will be used for reporting the trial. ^26^

The Standard Protocol Items: Recommendations for Interventional Trials (SPIRIT) Guidelines are used to report the study’s protocol. A completed SPIRIT checklist is one of the prerequisites of the reporting guidelines. ^27^

### Duration of the study

January 2023 - September 2024

### Sample size calculation

Assuming that the proposed intervention in this study will result in a reduction in the mean screen-based sedentary time by 0.9 hours (54 minutes) between intervention and the control groups, with 80% power and at a level of significance of 5%, the sample size was calculated to be 180 in each of the intervention and control arm. A design effect of 2 was assumed since this was a cluster randomized trial, and a sample size of 360 was determined for both the intervention and control groups. The final sample size was 400 in each arm, taking into account a maximum loss of 10% throughout the six-month follow-up period.

The sample size was calculated using the following equation ((1):

$$N=\frac{2\left[Z_{1-\frac{a}{2}}+Z_{1-\beta }\right]\sigma 2}{d^{2}}$$ (1)

Where,

*Z*_1- *α*/2_ = 1.96, Standard normal value at 5% level of significance

*Z*_1- *β*_ = 0.84, Standard normal value at 80% power

*σ* = standard deviation = 3.025 hours ^15^

*d* = clinically significant difference = 0.9 hours

### Eligibility criteria

All the students of class 8 of the selected schools in the urban area of Mangalore will be eligible to participate in the study.

### Study participants

Students studying in 8 ^th^ standard (middle school) in the selected schools in the urban area of Mangalore. The study population will be restricted to the students of 8 ^th^ standard since the students will be available in the selected schools for the entire duration of the study and loss to follow up is nil or minimal. Since the study has one year duration of follow up, students from 10 ^th^ standard will not be selected since they will complete their studies and shift to a different college.

### Exclusion criteria

Students whose parents do not give consent for their ward to participate in the study, students who do not assent to participate in the study and students who are not present on the day of baseline evaluation will be excluded from the study.

### Randomization technique

The list of schools situated in Mangalore will be obtained from the Block Education Officer/Deputy Director of Public Instruction. A comprehensive list of all eligible schools i.e., schools with classes 8 and above will be prepared. All the schools located within the limits of Mangalore City Corporation will be considered clusters. The study will employ cluster randomization, with clusters being allocated in a 1:1 ratio to the intervention arm and control arm. Randomization of schools will be done to accommodate 10 schools each in the intervention and control arm by simple randomization technique.

### Sampling technique

The study participants will be selected from the 8 ^th^ standard of selected schools. In schools with multiple sections of the same class, a simple random sampling technique will be employed to select one section, assuming a batch size of 40 students in each section. If the batch selected after simple random sampling has less than 40 students, the next batch will be selected by simple random sampling without replacement method, to reach the desired sample of 40 from the clusters.

The selected schools in the intervention arm would receive the comprehensive multicomponent modular intervention and the control group would not receive any intervention from the investigator. The students in the control group will undergo routine physical health education as per the school curriculum. They will be offered the comprehensive modular intervention only after the end of the final data collection.

### Multi-component modular intervention

The intervention designed will be a combination of a health education session, distribution of information flyers, and an activity designed to promote self-motivated behaviour change.

Based on suggestions from subject experts, a thorough literature assessment, and interaction with local stakeholders, a health education module will be created. This will be a multi-component educational module in English containing pertinent textual and illustrated/pictorial information curated according to the local context. The module has four main components:

• **Component 1: Sedentary behaviour**
  This will provide information as to what constitutes sedentary behaviour and the various activities which are considered sedentary in various settings including school and at home.
• **Component 2: Screen-based and non screen based sedentary behaviour**
  This will introduce the participants to the concept of screen time, and screen based and non screen based sedentary time. The behaviours constituting them will be explained using pictures and real life examples.
• **Component 3: Effects of sedentary behaviour**
  This component will explain to the participants the harmful effects of excessive screen time and how screen time is associated with reduced physical activity and sedentary behaviour, unhealthy eating and poor quality sleep. The section will also introduce the participants to the health effects of sedentary behaviour on the body and mind.
• **Component 4: Reducing screen time and sedentary behaviour**
  This component will introduce participants to various methods designed to reduce screen time like setting screen time limits for a day, turning off notification sounds in their phones to avoid picking up their phone more often, practising screen-free time e.g. avoiding screen use during lunch breaks, avoiding using screens before going to sleep, encouraging a screen-free bedroom, avoiding the use of eBooks to read novels, and taking part in screen-free physical activities that they enjoy to promote a healthy lifestyle. Since classroom learning can account for the least active time of a child’s day, incorporating physically active learning in between classes can help in reducing the amount of time children spend sitting down each day. The component will introduce the students to the concept of ‘movement breaks’ where the students will be taught how to break continuous periods of sedentary behaviour during the school hours. They will be taught small activities that they can do while still inside the classroom that we call ‘chairobics’. They will also be taught about how movement helps to increase blood flow around the body and to the brain so that they can feel alert, concentrate better and are ready to learn and think during the class.

The selected schools in the intervention arm would receive the multi-component modular intervention. Along with the educational module, a flyer depicting the effects of excessive screen time, risks of sedentary behaviour and tips for reducing screen time will be distributed to all the students in the selected class. Each student will also be given a notebook to log the duration of screen time during school and non-school hours (screen time tracking) duration of physical activity and duration of sleep until the end of the intervention period. At the end of every week’s recording, students will be encouraged to write a reflection on challenges or difficulties they faced in following a healthy lifestyle and how to overcome them.

### Intervention procedure

The class size of the selected schools is expected to be 40. The educational intervention will be delivered by the primary investigator. Three sessions at two monthly intervals will be conducted in the schools (Baseline, 2 ^nd^ month and 4 ^th^ month) selected to receive the modular educational intervention. The duration of each session will be 40 minutes.

The students in the schools selected for the control arm will not receive any intervention from the investigators. They will continue receiving the physical activity routine as per the school curriculum. The school will be offered the modular intervention only at the end of the final data collection.

### Operational definitions

**Screen time:** This refers to how much time a person spends using screen-equipped electronic devices, such as computers, televisions, game consoles, cell phones, and tablets, irrespective of whether the activity is carried out when physically active or sedentary. ^28^

**Screen-based sedentary time:** This refers to the duration an individual spends engaged in sedentary activities while using electronic devices with screens in any context (e.g., school, work, recreation). Sedentary activities involve minimal physical movement or energy expenditure and typically involve sitting or lying down for extended periods. ^28^

**Recreational screen time:** This refers to the period during which an individual engages in screen-based activities for leisure, entertainment, or personal enjoyment. This includes the time spent using screen-equipped electronic devices, such as computers, televisions, game consoles, cell phones, tablets, and other similar devices, for activities that are not work or education-related. ^28^

**Non-screen-based sedentary time:** This refers to the amount of time spent engaging in sedentary activities that don’t use screens. ^28^

### Instruments used for data collection

A pre-tested, content-validated semi-structured questionnaire will be used for data collection. The questionnaire was prepared after an extensive review of the literature and consultation with subject experts, and will include the following sections:

**Section 1:** Socio-demographic details

**Section 2:** HELENA (Healthy Lifestyle in European Adolescents) Screen-Time-Based Sedentary Behaviour Questionnaire ^29^

**Section 3:** Non-Screen-Based Sedentary Behaviour Questionnaire

**Section 4:** Physical Activity Questionnaire for Adolescents (PAQ-A) ^30^

**Section 5:** Anthropometric measurements based on STEPS questionnaire ^31^

### Healthy Lifestyle in European Adolescents Screen-Time-Based Sedentary Behaviour Questionnaire (HELENA) ^29^

HELENA questionnaire will be used to assess the screen-associated sedentary behaviour among the adolescents. The questionnaire requires adolescents to report how much time they typically spend on a variety of sedentary activities on weekdays and weekends.

The sedentary activities captured by the questionnaire include:

(i) Watching television
(ii) Playing games on the computer
(iii) Playing games on a console
(iv) Academic web browsing
(v) Nonacademic web browsing (E.g., Watching videos on YouTube)
(vi) Browsing through social media (such as Facebook, Instagram, etc.)

Depending on the time spent on these activities, the participants will choose one from the following options: (i) 0 hours (ii) < 1/2 hr (iii) ½ -1 hr (iv) 1-2 hrs (v) 2-3 hrs (vi) 3-4 hrs (vii) >4 hrs. Weekly time will be calculated by taking the mean time in the selected category and applying the formula: [(weekdays x 5) + (weekend x 2)] /7. A final screen-based sedentary score will be generated by adding the time reported for each category. ^29^

### Non-Screen Based Sedentary Behaviour Questionnaire

This section of the questionnaire was prepared after an extensive review of the literature. It captures the duration of time spent by adolescents during school days and holidays, carrying out sedentary activities which do not involve the use of screens. The activities included are as follows:

(i) Educational purposes: (number of hours sitting in the class, hours spent studying, doing homework, tuition class outside of school)
(ii) Recreational purposes: (hours spent sitting and playing games such as chess, carrom board, playing a musical instrument, hours spent on hobbies while sitting down such as reading novels, painting, arts and crafts etc)
(iii) Travel purposes: (hours spent in car, bus, school van etc.) and social purposes (talking with friends and family).

Depending on the time spent on these activities, the participants will choose one from the following options: (i) 0 hours (ii) < 1/2 hr (iii) ½ -1 hr (iv) 1-2 hrs (v) 2-3 hrs (vi) 3-4 hrs (vii) >4 hrs. Weekly time will be calculated by taking the meantime in the selected category and applying the formula: [(weekdays x 5) + (weekend x 2)] /7. The time reported for each category will be added up to produce a final non-screen-based sedentary time.

### Physical Activity Using the Physical Activity Questionnaire for Adolescents (PAQ-A) ^30^

It is an 8-item self-report questionnaire developed to assess the adolescents’ levels of physical activity over the previous 7 days. Each of the eight (PAQ-A) questionnaire items is scored between 1 and 5, and the mean score across all items is the total PAQ score. A score of 1 indicates low physical activity, whereas a score of 5 indicates high physical activity.

### World Health Organization (WHO) Stepwise Approach to Surveillance (STEPS) ^31^

This is a structured process for gathering, examining, and sharing information on noncommunicable diseases (NCDs) and their risk factors. Anthropometry measurements will be taken as per the standard and protocol outlined in WHO-STEPs.

### Data collection

The Institutional Ethics Committee (IEC) of Kasturba Medical College, Mangalore, has accepted the protocol (IECKMCMLR-02/2023/57). The trial has been registered with the Clinical Trial Registry-India (CTRI) (REF/2023/04/066280). Before the commencement of the study, the Block Education Officer, Mangalore will be approached to obtain the list of schools in Mangalore and to get the necessary permission to conduct the study in schools.

Permission to conduct the study in the selected schools will be obtained from the school administration. The school administration/Principal will be told the objectives of the study. On receiving the formal approval, the schools will be visited on a previously informed date. The study will be carried out among the students studying in class 8 of the selected schools. In case there are multiple sections for the same class, one of the sections will be selected for the study using simple random sampling.

Since the participants in the study are under the age of 18 years, documented parental consent will be required before their enrollment in the study. The information sheet and consent form will be distributed to the students of the selected class on the first visit to be shared with their parents. On the second visit, a written assent will be taken from the students whose parents have consented and the questionnaire will be distributed.

### Baseline data collection

A self-administered questionnaire will be used to gather the demographic data, which includes information like age, gender, class, section, address, contact information, and the parents’ or guardians’ occupations. The study participants will be asked about their screen- and non-screen-based sedentary time as well as their physical activity. Anthropometric measurements like height, weight, waist and hip circumference will be collected using standardized techniques. ^31^

**Figure 1.**
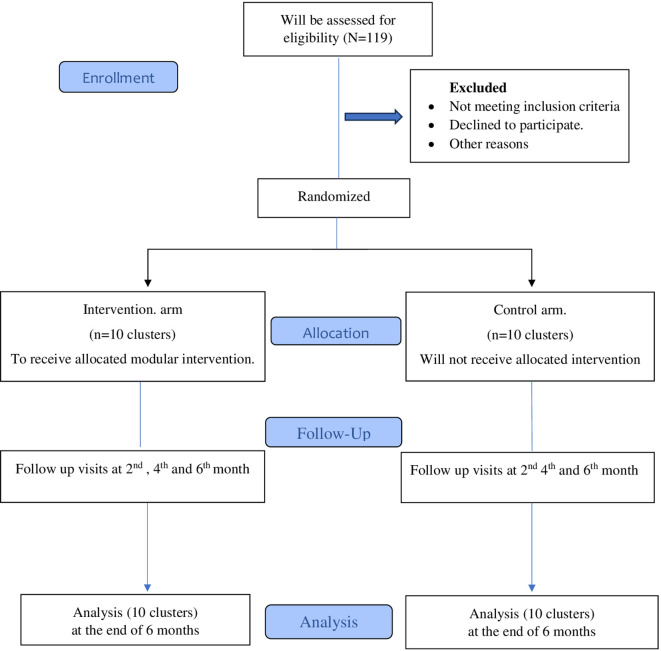
Study CONSORT diagram.

Following the collection of baseline data, the schools selected for intervention will receive the multi-component modular intervention. Three sessions at two monthly intervals will be conducted in these schools. (Baseline, second month and fourth month) Both groups will complete the questionnaires at baseline, and two, four and six months of follow-up. Anthropometric measurements will be taken at baseline and sixth month of follow-up) Outcome will be assessed at each point of time in both intervention and control arm students. Confidentiality and anonymity will be maintained throughout the study ( Figure 1).

### Procedure for anthropometric measurements

**Measurement of weight**

A digital weighing scale will be used to measure weight. The patient will be requested to take off their shoes, socks, and any additional footwear they may be wearing before being instructed to place one foot on either side of the scale. The participant will be instructed to stand still, with their arms at their sides and their face front. The scale will record the weight in kilos to the nearest 0.1 cm. After each use, the scale will be reset to zero. ^31^

**Measurement of height**

A portable stadiometer will be used to measure the height. The participant will be directed to take off their shoes, slippers, and sandals in addition to their hat, cap, hair bows, comb, and ribbons. The participant will be instructed to face the investigator and stand on the board. He/She will be instructed to stand with their feet together, knees upright, and heels touching the backboard. He or she is instructed to maintain a straight-ahead gaze and to keep their eyes level with their ears. By lowering the measuring arm to the patient’s head, the reading will be measured to the nearest 0.1 cm. ^31^

**Body mass index** ^32^

Body Mass Index (BMI) will be calculated using the equation (2):

$$\mathrm{BMI}=\frac{\text{Weight}\;\left(\text{in}\;\mathrm{kg}\right)}{\text{Height}\;{\left(\text{in meter}\right)}^{2}}$$ (2)

The presence of being overweight and obesity will be assessed by computing BMI for age z scores and comparing them to the age- and sex-specific BMI z score +1 cut-offs published by the World Health Organization. ^30^ The interpretation of BMI z scores is as follows: The presence of being overweight and obesity will be assessed by computing BMI for age z scores and comparing them to the age- and sex-specific BMI z score +1 cut-offs published by the World Health Organization. ^30^ The interpretation of BMI z scores is as follows:

Overweight: >+1 SD (equivalent to BMI 25 kg/m ^2^ at 19 years)

Obesity: >+2 SD (Equivalent to BMI 30 kg/m ^2^ at 19 years)

**Measurement of waist circumference**

Waist circumference will be taken halfway between the bottom of the last rib and the top of the iliac crest (hip bone), at the end of breathing, with arms at the sides using a measuring tape. The subject is instructed to put the tape over themselves. It will be instructed for the subject to stand with their feet together, their weight evenly spread over both feet, and their arms relaxed by their sides. Only one measurement will be made to the closest 0.1 cm. ^31^

**Measurement of hip circumference**

Hip circumference will be calculated using a measuring tape. The participant must stand with their feet together, their weight evenly distributed over both feet, and their arms at their sides. The tape would be applied to the buttocks’ largest circumference. Measurements will be made to the nearest 0.1 cm at the level of the tape. ^31^

### Outcome variables

1. Mean screen sedentary time (SST) and non-screen sedentary time (NSST)
2. Level of physical activity
3. Association of BMI, waist circumference hip circumference and waist height ratio with screen sedentary time and non-screen based sedentary time
4. Difference in the mean SST and mean NSST between the intervention and control group

### Data management

Utilizing IBM SPSS (Statistical Package for Social Sciences) Statistics for Windows Version 25.0, the gathered data will be coded, inputted, and analyzed. Armonk: IBM Corporation. ^33^ The proformas will be checked for completeness and the participant will be contacted for any missing information. The data will be kept confidential and protected using encryption.

### Data analysis

Proportions will be used to express the results. The interquartile range (IQR) and standard deviation (SD) associated with summary measures like mean and median will be reported for continuous variables. ITT analysis—intention to treat—will be used. The data will be represented by suitable tables and figures. The chi-square test will be used to compare variables between intervention and control groups. The baseline and post-intervention values across the intervention and control groups will be compared using the Mann-Whitney U test for non-normal data. (ST, SST, NSST) For data following normal distribution (WC, HC) independent t-test will be used and p < 0.05 will be considered to be statistically significant. Repeated measures of ANOVA will be used to compare the change scores (ST, SST, NSST) within the intervention and control groups and ’a p-value less than < 0.05 will be considered statistically significant.

### Implications

A comprehensive school-based modular intervention with an active behavioural change component like the one utilized in this study can have cumulative advantages by reducing screen- and non-screen-based sedentary time, encouraging physical activity, and promoting quality sleep to mitigate each factor’s individual effects on adiposity measurements in teenagers. It can be effective in promoting healthier habits and reducing the negative impacts of excessive screen time on academic performance, physical health, and overall well-being.

### Ethical considerations

The Institutional Ethics Committee (IEC) at Kasturba Medical College, Mangalore has approved the study protocol.

If changes are made to the protocol after the commencement of the study, it will be resubmitted to the IEC for approval.

The trial has been registered with the Clinical Trial Registry-India (CTRI) (REF/2023/04/066280).

Required permissions will be obtained from the block educational officer and the school administration.

Written parental approval and informed assent will be obtained from the participants. The participant information data will be handled with the utmost confidentiality.

### Data monitoring

An interim analysis will be carried out after 3 months from the commencement of the study. There is no known adverse effect associated with this study. There will not be any committee formed for data monitoring. The selected schools in the control arm will be offered the modular intervention at the end of the final data collection.

### Dissemination

Presentations at scientific meetings and publications will be used to publicize the study’s findings. As stated in the methodology, we will report the study’s flow and results using the CONSORT principles. The Standard Protocol Items: Recommendations for Interventional Trials (SPIRIT) Guidelines are used to report the study’s protocol. As a result of the research data’s potential to compromise participants’ anonymity and the fact that they contain personal information, the IEC guidelines of the study institute forbid sharing research data with any other organization. However, the information can be obtained from the associated author upon personal request and with sufficient cause.

### Current status of the study

The study has not started recruiting participants.

## Data availability

No data are associated with this article.
