# Supplementary material for: Effectiveness of multi-component modular intervention on screen-based and non-screen-based sedentary time among adolescents in an urban area of Mangalore: a school-based cluster randomised controlled trial-protocol
Source: F1000Res. 2024 Mar 11;13:70. Originally published 2024 Jan 15. [Version 2] doi: 10.12688/f1000research.142350.2 (PMC10958146; doi:10.12688/f1000research.142350.2)
Supplement: Peer review history

# Reviewer report: Reviewer response for version 2

**DOI:** 10.5256/f1000research.163553.r254563

**Published:** 2024-03-26

**Authors:** Sudhir Prabhu H

**Affiliations:** 1 Department of Community Medicine, Father Muller Medical College, Mangalore, India

The changes incorporated are satisfactory

Is the study design appropriate for the research question?

Yes

Is the rationale for, and objectives of, the study clearly described?

Yes

Are sufficient details of the methods provided to allow replication by others?

Yes

Are the datasets clearly presented in a useable and accessible format?

Yes

Reviewer Expertise:

Immunization, Public health

I confirm that I have read this submission and believe that I have an appropriate level of expertise to confirm that it is of an acceptable scientific standard.

# Reviewer report: Reviewer response for version 2

**DOI:** 10.5256/f1000research.163553.r254564

**Published:** 2024-03-21

**Authors:** Jay Sheth

**Affiliations:** 1 Department of Community Medicine, Narendra Modi Medical College, Maninagar, Ahmedabad, India

Yes, the authors have addressed most of the concerns raised in the first review. I have no further major concern related to the article

Is the study design appropriate for the research question?

Yes

Is the rationale for, and objectives of, the study clearly described?

Yes

Are sufficient details of the methods provided to allow replication by others?

Partly

Are the datasets clearly presented in a useable and accessible format?

No

Reviewer Expertise:

Public health, epidemiology, research methodology, NCDs

I confirm that I have read this submission and believe that I have an appropriate level of expertise to confirm that it is of an acceptable scientific standard.

# Reviewer report: Reviewer response for version 1

**DOI:** 10.5256/f1000research.155892.r238235

**Published:** 2024-02-20

**Authors:** Jay Sheth

**Affiliations:** 1 Department of Community Medicine, Narendra Modi Medical College, Maninagar, Ahmedabad, India

In the abstract, the first line should have ‘may’ instead of ‘can’

 Many sentences in the introduction, which have some numbers or major scientific documented facts, do not have any reference. Ideally, it has to be supported by appropriate recent and authentic references.

 There is some duplication of information (e.g. related to risk factors of NCD) which should be avoided for a smooth flow of text and better understanding.

 As the study aims to decrease SST and NSST, a hypothesis could have been added in the protocol.

 Knowledge gaps are well identified but some of the gaps are going beyond the focus and scope of the protocol. The authors may focus more on those gaps which the present protocol would be addressing scientifically.

 Objective1, Objective 2 & Objective 3 focuses on baseline, developing a modular intervention and Assessing its effectiveness in sequence. Here, after development, implementation of the multicomponent modular intervention may be added between objective 2 & objective 3. Alternatively, objective 2 may have “Develop and implement” as the action verb.

 There is some difference between “eligibility criteria”, “study population” and “inclusion criteria”.

  What the authors have suggested after the first line under “eligibility criteria” are actually exclusion criteria. The flow should be “eligible criteria” followed by “study population”, followed by inclusion criteria and then the exclusion criteria.

 The protocol assumes a batch size of 40 students for standard 8 of each school. However, if there are less than 40 (available, gives consent and enrolled) what is to be done to achieve the desired sample of 40 from the cluster, should have been included in the protocol. As this is a protocol, the authors should refrain from any assumption and prepare it thorough to address all possibilities, only then it can be easily adopted and replicated by others.

 Components of multicomponent Modular intervention are clarified. Component 4 lists multiple activities and action points, however, it gives only an idea of the broad gamut it covers and conveys. It might be possible that this component is delivered differently in different clusters and by different investigators. Authors may consider streamlining and standardizing the component so that all investigators in all the intervention cluster apply it in a uniform manner. If this can be added and clarified, it will greatly improve the standard of intervention.

 Whether component 1,2 & 3 will be given but component 4 will not be addressed in control clusters? If there is no intervention in the control arm cluster, what the investigators will do there during follow up (after baseline)? I am unable to clearly understand this aspect.

 Instrument used for data collection is not available here. Without its availability detailed review of the tool is not possible.

 Since it's not a blinded study and intervention (educational intervention) is obvious, whether permission from school authorities (for the control cluster group) will be possible? Consider why and how the schools will give permission to be included in the control cluster. Even if they are not told, they will know that they are part of control cluster if nothing is done apart from bimonthly data collection after the baseline.

 Interim analysis at 3 month seems to be misfit. Why it can't be done or should not be done at first follow up at 2 months? What is the specific advantage of doing it at 3 months at the cost of an additional visit?

 This is a protocol so dataset may not be applicable. But, study tool and details of multicomponent module may be included.

Is the study design appropriate for the research question?

Yes

Is the rationale for, and objectives of, the study clearly described?

Yes

Are sufficient details of the methods provided to allow replication by others?

Partly

Are the datasets clearly presented in a useable and accessible format?

No

Reviewer Expertise:

Public health, epidemiology, research methodology, NCDs

I confirm that I have read this submission and believe that I have an appropriate level of expertise to confirm that it is of an acceptable scientific standard, however I have significant reservations, as outlined above.

# Reviewer report: Reviewer response for version 1

**DOI:** 10.5256/f1000research.155892.r238233

**Published:** 2024-02-09

**Authors:** Sudhir Prabhu H

**Affiliations:** 1 Department of Community Medicine, Father Muller Medical College, Mangalore, India

The research question addresses a very important public health issue of concern and the study design being an interventional study will be apt in addressing it. The Study tool used are extensive and comprehensive for addressing the research objectives. However, there are a few scientific and ethical concerns in the protocol. 

 The scientific concerns to the protocol submitted include:

 1. Why the study population is being restricted to 8th std students only? Any specific reasons can be mentioned in methods section.

 2. Will there be adequate representation of primary sampling units in the school-based cluster (dual medium, dual lingual, type of curriculum, type of board etc.)?

 3. A statement on how will screen based sedentary and non-screen based sedentary time be explained to students can be mentioned (since it's the primary objective of the study)

 Ethical concern:

 A statement on informed parental consent being taken and its process can be mentioned.

Is the study design appropriate for the research question?

Yes

Is the rationale for, and objectives of, the study clearly described?

Yes

Are sufficient details of the methods provided to allow replication by others?

Yes

Are the datasets clearly presented in a useable and accessible format?

Yes

Reviewer Expertise:

Immunization, Public health

I confirm that I have read this submission and believe that I have an appropriate level of expertise to confirm that it is of an acceptable scientific standard.
